# Accelerated annealing of fused filament fabricated (FFF) thermoplastics via an improved core–shell filament

**DOI:** 10.1038/s41598-023-40855-6

**Published:** 2023-08-19

**Authors:** Michael Pugatch, Molly Teece, Juhyeong Lee, Nikhil Patil, Ryan Dunn, Kevin Hart, Eric Wetzel, Jay H. Park

**Affiliations:** 1 Department of Plastics Engineering, University of Massachusetts-Lowell, Lowell, 01854 USA; 2 United States Army Research Laboratory, Aberdeen Proving Ground, MD 21005 USA; 3 Milwakuee School of Engineering, Milwaukee, WI 53202 USA

**Keywords:** Polymers, Mechanical engineering

## Abstract

Thermoplastic parts manufactured via fused filament fabrication (FFF) have limited strength and toughness compared to other types of polymer additive and subtractive manufacturing. Low strength results from poor interlayer adhesion, making FFF parts not suitable for most engineering applications. Post processing solutions, such as annealing, enable healing of these interlayers, thus approaching injection molded parts. Prior work demonstrated a core–shell polycarbonate (PC)—acrylonitrile butadiene styrene (ABS) structured dual material filament to provide thermo-structural stability during annealing of the ABS component; however, annealing was limited to relatively low temperatures (135 °C) and required long annealing times (72 h). In the current work, a PC copolymer with a higher glass transition temperature (173 °C) than conventional PC is processed along with an extrusion-grade ABS into a PC-ABS core–shell filament. This improved dual material filament was printed, annealed, and evaluated via Izod impact testing, ultimately yielding 83% of bulk annealed ABS z-direction strength at an accelerated annealing time (8 h) and higher annealing temperature (155—175 °C). A demonstration part is printed with the dual material filament and annealed at 155 °C for 8 h, resulting in excellent dimensional accuracy, and a ductile failure at 73% higher ultimate load compared to the brittle failure of an as-printed part. This work highlights that material selection and design of a bicomponent filament geometry can lead to parts printed with FFF, with increased strength compared to other post-processing techniques at reduced processing times.

## Introduction

Fused filament fabrication (FFF) is a well-known additive manufacturing method in which thermoplastic material is melted and extruded layer-by-layer, capable of rapidly prototyping complex parts that may not be possible using other manufacturing methods such as injection molding^1^. While originally considered a prototyping tool, the technology has advanced from simple jigs and fixtures to intricate functional parts as well as a primary manufacturing method for parts with complex or bespoke geometric forms^1,2^. While FFF-printed parts can function and hold tight tolerances, they are not widely considered reliable for long-term engineering applications that require mechanical resiliency. The print build or “Z” direction exhibits low strength relative to bulk polymer properties due to poor layer-to-layer adhesion. Prints are formed layer-by-layer where the active, molten layer is deposited over a partially cooled under-layer, which limits polymer chain diffusion and produces a significantly weaker interface between each layer than in the bulk of the extrudate trace^3–5^. To improve interlayer strength, print settings can be optimized^5^, modifications can be made to the printer^6^, in-situ^7,24^ or post-print processing^8^ can be implemented, or the filament itself can be specially engineered^9^. Among several post processing methods, annealing is effective at healing these interfaces between print layers^3^.

Other researchers have studied ways to enhance the functionality and resolution of FFF-printed parts. Butt and Bhaskar^10^ looked at the effect of free-standing annealing on mono-thermoplastics parts made of PLA and ABS where they observed an increase in the tensile strength but noted the adverse effect of annealing on dimensional tolerances. Bhandari et al*.*^4^ employed annealing to achieve a 17% increase in tensile interlayer strength of extrusion 3D printed PLA, and a 64% increase in strain to failure of carbon fiber reinforced PETG. Dunn et al*.*^11^ evaluated a dual-material print schematic in which two mono filaments were used to print a bulk ABS core and encasing PC shell, which acted as a mold during annealing. They achieved a 90% increase in ultimate load on the annealed parts while also retaining part geometry. Ai et al*.*^12^ evaluated a core–shell filament architecture with polycarbonate core to address dimensional accuracy. They found that the impact resistance was linked to the material selection of the core polymer, and demonstrated how the core–shell architecture helps mitigate the trade-offs between mechanical properties and dimensional stability in FFF by maximizing the difference in solidification temperature between the core and shell materials. Core–shell architecture has also been studied in different geometric configurations, such as in Hart et al*.*^9^, which determined an asterisk-shaped core fully encased by the shell to be optimal for achieving toughness post-annealing. They established that interlayer fusion in FFF-printed parts relies on four critical steps: interfacial molecular heating, contact, diffusion, and cooling.

The necessary annealing conditions for printed amorphous thermoplastic structures are best determined relative to the polymer’s glass transition temperature (T_g_). Above the T_g_, surface tension drives an increase in wetted contact area between layers, which enables polymer reptation and formation of a well-healed, strong layer-to-layer interface. Hart et al*.*^13^ determined that wetting of the polymer melt is the rate-limiting step for thermal annealing of amorphous thermoplastics. A challenge during thermal annealing is maintaining accurate part geometry while annealing above the polymer T_g_. To thermally anneal thermoplastic FFF-printed parts, sufficient molecular motion of the micro-structure must be induced; however, under this condition, macroscopic creep and loss of dimensional accuracy is also likely.

One method to improve geometric integrity during annealing is the implementation of a core–shell filament architecture, in which a core material of higher thermal stability than the shell imparts structural stability to the system during annealing^9,14^. In these prior studies, annealing times of 72 h were necessary to achieve high strength. Annealing temperatures were kept well below the T_g_ of the core material to ensure geometric stability during annealing. For many applications, such as on-demand manufacturing, reducing this annealing time would be beneficial for reducing costs and achieving faster production. Establishing a faster method to improve the mechanical properties of FFF-printed thermoplastic parts helps bridge the gap to mechanically superior, injection molded thermoplastic parts.

Using a higher T_g_ core material than previous works^9,14^ should allow for increased annealing temperatures, reducing the overall effective annealing time by accelerating wetting and reptation for the shell material. In this work, we evaluate a series of dual material core–shell filaments using two different grades of acrylonitrile–butadiene–styrene (ABS) in the shell, and two core materials: a conventional polycarbonate (PC), and a PC copolymer with an elevated glass transition temperature. Our new approach is to use a high T_g_ polycarbonate core intended to enable thermo-structural stability during annealing at higher temperatures, thereby reducing annealing time compared to parts supported by conventional PC with lower T_g_, such as those studied in Hart et al. ^9^ and Koker et al.^14^. The ABS shell, on the other hand, acts as the wetting layer between the print lines and layers to enhance mechanical properties post-annealing. The aim of this study is to meaningfully reduce the overall annealing time for FFF-printed thermoplastic parts while achieving an increase in mechanical properties comparable to previous works^9,14^.

## Results

### Mechanical and thermal assessments of single and dual-material prints

**Figure 1:**
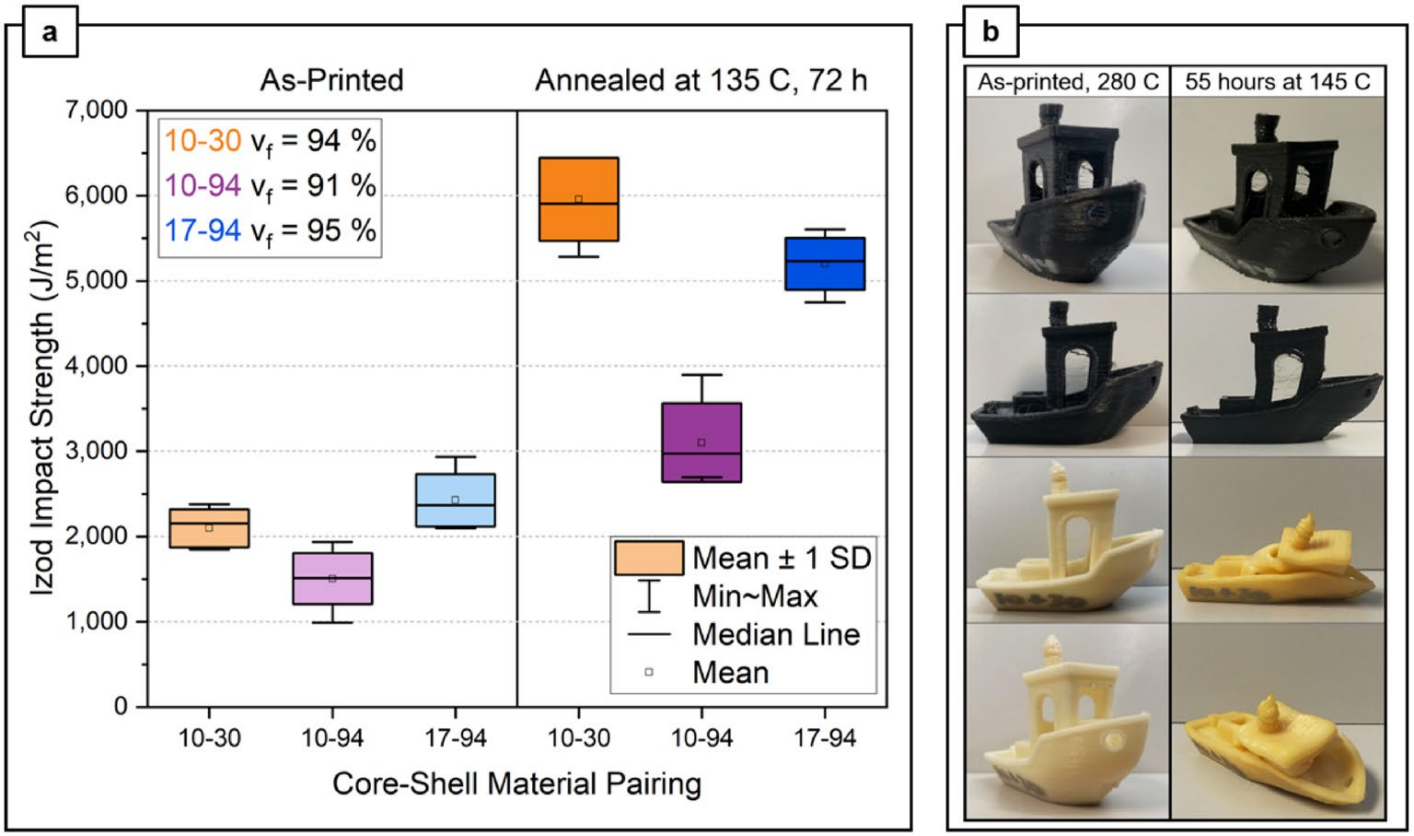
(**a**) As-printed and annealed Izod impact data for different filament compositions. (**b**) Geometric stability comparison of 17–94 (top four, black Benchy) and 10–30 (bottom four, white/yellow Benchy), before and after annealing for 55 h at 145 °C.

Dual material filaments with core–shell combinations of 10–30, 10–94, and 17–94 were printed and tested for Izod impact strength, repeated for as-printed and annealed samples (Fig. 1). Measured print fill fractions were ${v}_{f}$ = 94%, 91%, and 95%, respectively. Among the as-printed samples, the order of impact strength from low to high is 10–94, 10–30, and 17–94. 10–94 and 10–30 are statistically comparable, as is 17–94 with 10–30. 10–94 and 17–94, on the other hand, are statistically different; the filament with higher T_g_ PC has led to higher Izod impact strength overall. In general, annealing enabled Izod impact strength increases across 10–30 (284%), 10–94 (200%), and 17–94 (214%). As with Koker et al.^14^, the specimens were annealed at 135 °C for 72 h, which is below the T_g_ of both polycarbonate grades and above T_g_ of the ABS grades used; no samples deformed after annealing. Among the annealed samples, 10–30 yielded the highest impact strength, followed by 17–94, although the results are statistically indistinguishable between the two. It is notable that the Izod impact strength of annealed M30 is around 12,000 J/m^2^ ^14^, while that of annealed MG94 yielded 6,066 J/m^2^ (v_f_ = 94%). Lastly, 10–94 after annealing exhibited the lowest Izod strength, presumably due to the combination of ABS with lower Izod impact strength and lower T_g_ PC. Implications of these results are further explored in the discussion section.

DSC confirmed the T_g_ values of Apec 1795 PC (173 °C) and PC-10 (144 °C), while the T_g_ values of both ABS grades are nearly identical at 107 °C. The T_g_’s of all four evaluated materials are tabulated in Table 1. Benchy samples printed at 280 °C with MG94, 10–30, and 17–94 are annealed at 145 °C for 55 h (Fig. 1b), which were checked for visible deformation at 5 and 10 h. At 145 °C, the MG94 Benchy significantly deformed well before the first observation point of 5 h. At 10-h mark, minor deformation had occurred with 10–30, while 17–94 yielded no notable structural deformation. This deformation was seen to propagate at overhangs (windows, bow), followed by the buckling of the bridge. At 45 h, both 10–30 and MG94 Benchy samples completely collapsed, while only minor deformation was noted for 17–94. The largest degree of deformation of the 17–94 Benchy was the overhanging bow, which sagged by 5.9 mm after 55 h (end of test).

**Table 1:** Average T_g_ of materials used measured by DSC.

Material	T_g_ (°C)
PC-10	144
1795	173
M30	107
MG94	107

### 17–94 dual material print evaluation

The thermal and mechanical results clearly indicate that 17–94 dual material pair yields competitive Izod impact strength with significantly higher dimensional stability at T = 145 °C, slightly above T_g_ of PC-10. As such, 17–94 is further assessed for annealing at higher temperature at a faster time scale. 17–94 was thus assessed at 8 h of annealing under 135 °C, 145 °C, 155 °C, and 175 °C. These annealing conditions were selected based on the calculated time and temperature for fully annealing ABS-M30 from Hart et al. ^13^, which is then corrected for MG94 based on Supplementary Fig. S1.

**Figure 2:**
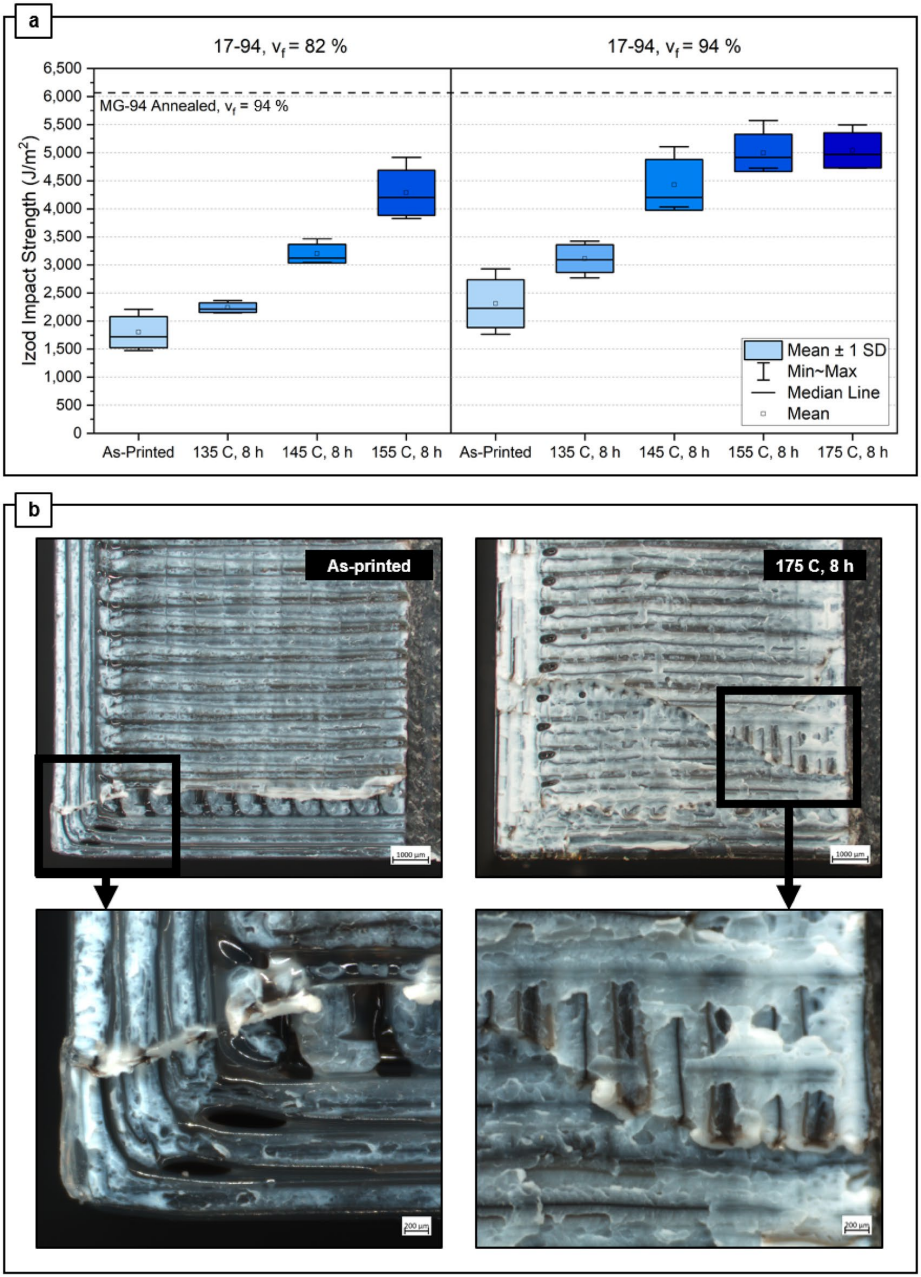
(**a**) Impact strength as a function of fill density (${v}_{f}$ = 82% and 94%) and annealing temperature for 17–94 filament, and reference line for printed ABS MG-94 annealed at 135 °C for 72 h. (**b**) Fracture surfaces of ${v}_{f}$ = 94% samples at ×12  and ×40 magnification.

Figure 2a shows the results of Izod impact testing; increasing annealing temperatures and higher fill density lead to higher impact strength. Samples were also assessed before annealing to confirm no critical print defects, and after impact testing to observe failure mechanisms. Figure 2b demonstrates the fracture surfaces across the different annealing temperatures. As the annealing temperature increases, the voids coalesce to form round air bubbles. Stress whitening is also observed in the ABS portion of the fracture interface. The coalesced voids and stress whitening are indicative of the polymer chain diffusion described in the section above and associated with adequate annealing. This is confirmed by the Izod impact strength results in Fig. 2a, where 83% of the Izod impact strength of ABS MG94 (${v}_{f}$ = 94%) annealed in-mold is achieved by annealing 17–94 filament free-standing at 155 °C or 175 °C for 8 h. This is a substantial improvement on the previous work, Koker et al*.*^14^, where the annealed parts printed with dual material achieved only 50% of the reference ABS-M30 annealed in-mold.

**Figure 3:**
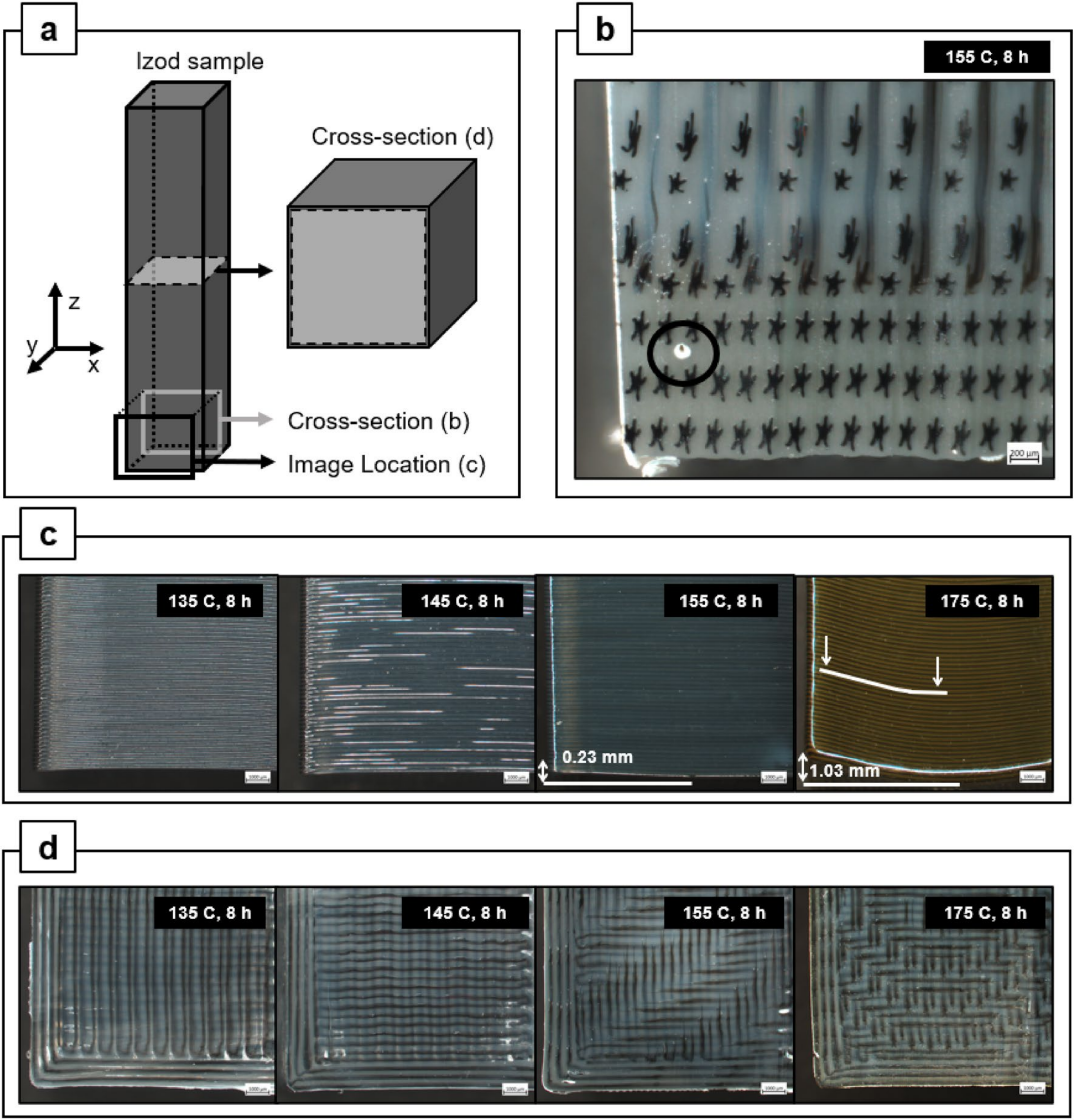
All specimens shown here are 17–94 with ${v}_{f}$ = 94%. (**a**) Schematic to show cross-sectional and imaging planes. (**b**) Cross section of X–Z plane showing core–shell configuration, and intersection of print perimeters with 0/90 raster infill where a single void is observed. (**c**) Surface finish and dimensional stability of 17–94 printed Izod bars as a function of annealing temperature, and (**d**) X–Y plane cross sections where print layers internally shift more with higher annealing temperature.

Figure 3a shows schematics of how Izod samples at ${v}_{f}$ = 94% were sliced and imaged for evaluation. Figure 3b displays a precision-cut cross-section of an Izod sample annealed at 145 °C for 8 h, where the intersection of the print perimeter lines and the 0/90 raster infill is shown, and a single void spot is observed. The relative shape and orientation of the asterisk core geometry shifts depending on the angle of the extrudate print path where the cross-section was taken, which is determined by the G-code slicing. Notably, the asterisk core was preserved during printing, consistent with prior works^9,14^. Figure 3c illustrates the changes in dimensional stability as the samples warped more at higher annealing temperatures, forming a gentle “U” shape. Figure 3d shows clean-cut cross sections of the samples in the XY plane (*i*.*e*., parallel to Z-axis interlayers) which shows how the center of the layers shifted in the Z-axis direction. This cut is perpendicular to Z, in which more layers are seen in the XY cross section of samples that were annealed at higher temperatures. The change in surface finish can also be observed in Fig. 3c, which is linked to the amount of wetting at the surface between the Z-axis print layers. The part surface becomes smoother and glossier as the annealing temperature increases, and superficial discoloration is observed in parts annealed at 175 °C. The observed change in surface gloss is the visual effect of the print layers flowing into one another, signaling that polymer chain diffusion and entanglement has occurred, which is necessary for effective bonding between extrudate lines in amorphous polymers processed via FFF, as noted in Ai et. al.^12^.

### Engineering application

**Figure 4:**
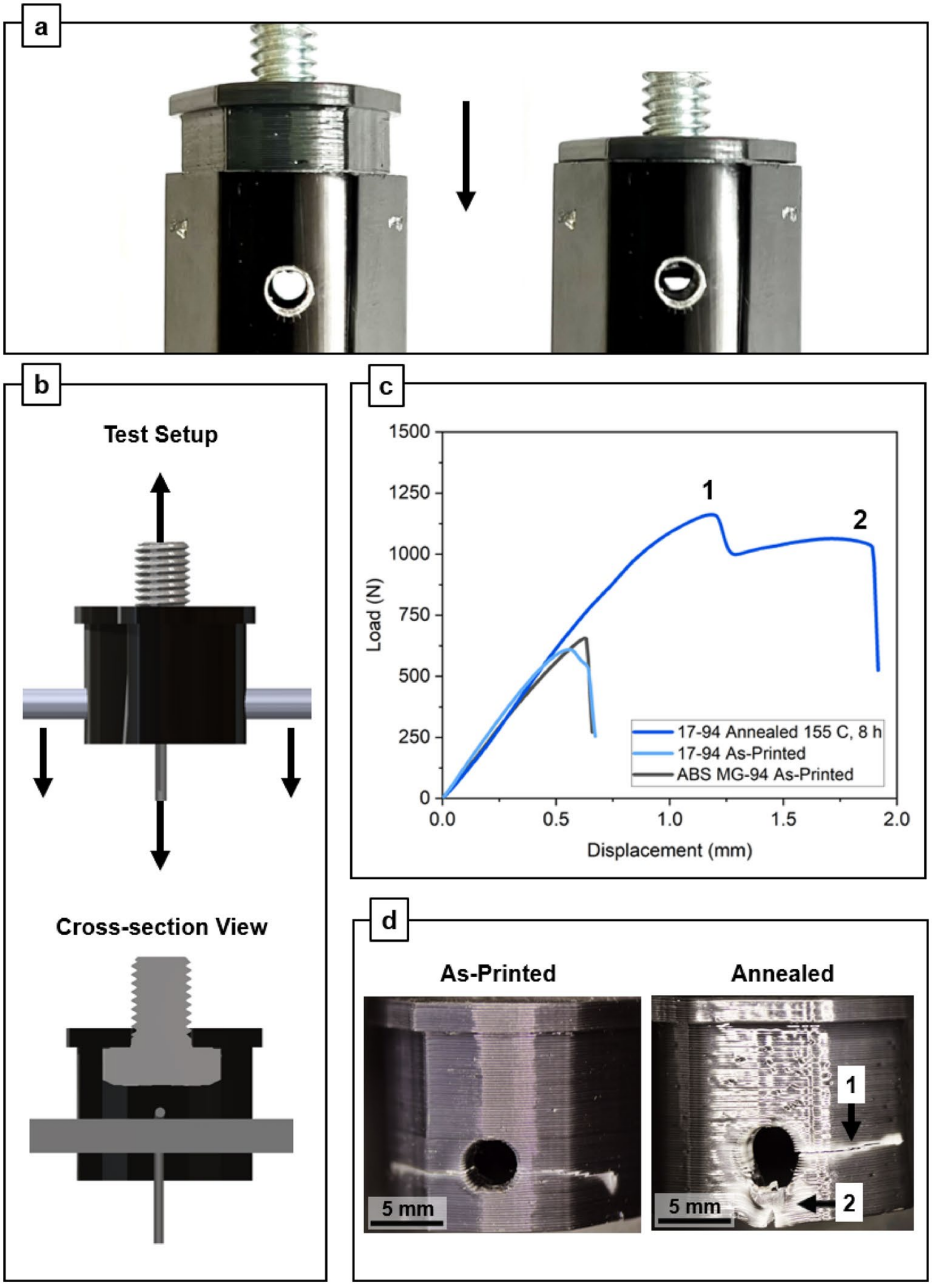
(**a**) Insertion of annealed part into tripod receiver, demonstrating dimensional accuracy. (**b**) Setup for part evaluation, (**c**) tensile results for exemplar samples, and (**d**) images of 17–94 as-printed and annealed fracture behavior, latter of which two yield points [correspond to 1 and 2 indicated in (**c**)] are observed in the annealed sample. All specimens are ${v}_{f}$ = 94%.

The thermomechanical advantages of the 17–94 filament are showcased in an engineering application by printing a functional part: a tripod camera mount adapter^19^. This part is not available as a replacement from the original equipment vendor, and the application requires robust material that can withstand the wear and tear of travel and heavy use with expensive, heavy camera equipment. All parts were printed using the parameters specified in Table 2. Five parts were printed using 17–94 filament and tested for each configuration: as-printed and annealed at 155 °C for 8 h. These annealing parameters were selected for the optimal combination of Izod impact strength, dimensional stability, and surface finish, which can be observed in Fig. 3c. Single material ABS MG-94 filament was also printed and tested as-printed. For clarity, one exemplar result for each sample configuration is shown; all tensile results are displayed in Supplementary Fig. S7. The annealed parts display excellent dimensional stability as there were no issues fitting the part into the intended assembly, which includes a press fit, seen in Fig. 4a.

**Table 2:** Print settings for all specimens.

Print setting	Value
Layer height	0.2 mm
Number of perimeters	3
Infill	100%
Raster pattern	0/90 alternating
Cooling fan	Off
Nozzle temperature	280 °C
Bed temperature	90 °C

The adaptors were tested using a modified tensile setup illustrated in Fig. 4b, designed to simulate the failure mode of the part if pulled out of the tripod assembly. A ¼-in x ½-in hex bolt was inserted into the part as shown in Fig. 4b, and a steel locking pin was then inserted horizontally. Finally, a steel wire bent into a U-shape was looped around the center of the locking pin and clamped into the lower tensile clamp. The protruding bolt was fixed into the upper tensile clamp, and all slack was removed from the system before testing. This test setup is intended to capture the complex combination of tension, compression, and bending that is experienced throughout the part volume. Figure 4c shows the results of this testing. Parts annealed using the recommended schedule of 155 °C for 8 h displayed failure loads of 1104 ± 55 N, compared to 612 ± 86 N and 663 ± 74 N for as-printed 17–94 and MG94 parts, respectively. Overall the annealed parts exhibited an average increase of 73% and 128% in ultimate failure load and elongation, respectively, over the as-printed parts. The load curves in Fig. 4c also show that as-printed parts fail in a brittle, catastrophic manner, whereas the annealed parts display a more gradual, graceful failure indicative of fracture toughness. An image of the full tripod mount assembly can be found in Supplementary Fig. S8.

## Discussion

Most processing difficulties with coextrusion were attributed to differences in melt temperatures between the core and shell materials. In the production of 17–94, the ABS shell was farther above its T_g_, requiring a longer time to cool. The procedure for cooling did not change across trials, hence slight ovality was imparted as the 17–94 filament was tensioned around the first roller. This ovality did not affect the standard deviation nor printability of the filament so long as the maximum filament diameter did not exceed 1.78 mm. Under this limitation, the average produced filament diameter was lower than the commercial standard of 1.75 mm, at 1.63 mm. However, the filament diameter was sufficiently consistent for printing, and the diameter value is automatically adjusted for in the print slicer software.

The increase in Izod impact strength after annealing is attributed to interlayer healing of the ABS. Between 17–94 and 10–94, the effects of incorporating PC with different T_g_ are observed. The higher Izod impact strength of 17–94 than 10–94 is consistent with the observation by Ai et al. where core–shell combinations with larger solidification temperature differences led to increased toughness^12^. While 10–30 almost triples in Izod impact strength after being annealed at the same conditions, 17–94 can be annealed at 20 °C higher for shorter amount of time, which is more economical due to shorter post-production time and less energy required. Moreover, annealed Benchy samples of 17–94 demonstrated far superior thermo-structural stability at elevated temperatures than the 10–30 pairing. This result is expected and attributed to the ~ 30 °C increase in T_g_ between 1795 and PC-10, and the annealing temperature of 145 °C is between the T_g_ of PC-10 and APEC 1795. The observation of some deformation for 17–94 parts at 155 °C was somewhat surprising, given that this annealing temperature is nearly 20 °C below the T_g_ of the core material. These deformations could be due to the very low viscoelasticity of the shell material at this high temperature. Increasing fill density to closer to 100%, as discussed below, may reduce these deformations and allow for practical annealing at or slightly above 155 °C.

Izod samples printed with 17–94 filament were evaluated at both 94% and 82% print density (Fig. 2a). At 82% print density, the dimensional stability of the parts drastically reduces; at 155 °C 8 h anneal condition, significant warping is observed for the 82% density sample. When these samples were annealed at 175 °C for 8 h, the warping was far more pronounced hence accurate Izod impact strength results could not be obtained and were therefore omitted. Annealed samples at ${v}_{f}$ = 82% collapsed inwards to form a trapezoidal shape: images can be seen in Supplementary Fig. S9. This deformation is theorized to be attributed to the lower print density and therefore increased instance of voids within the structure, which reduce the thermo-structural stability of the part. Increased instance of voids also drastically reduces the Izod impact strength of the parts since they act as stress concentrators^20–22^. For the ${v}_{f}$= 94% 17–94 samples, the specimens displayed no significant warping at any annealing temperature; however slight upward (in z-direction) deformation of 0.23 mm relative to the as-printed print face was observed at 155 °C for 8 h samples, and slightly higher deformation of 1.03 mm was observed for samples annealed at 175 °C for 8 h. The Izod impact strength increases with higher temperature and plateaus at 155 °C and 175 °C. These values are comparable to 17–94 annealed at 135 °C for 72 h (Fig. 1a), clearly demonstrating the effectiveness of the accelerated annealing timescale for 17–94.

The origin of enhanced impact strength with respect to temperature and time is investigated based on a sintering model of annealing^9^. From the temperature–time superposition and WLF fitting^23^ of ABS MG94 shell material (Supplementary Fig. S1), one can derive the following characterization time for complete wetting of ABS:

1 $${t}_{s}=3\frac{{a}_{0}\upeta }{\Gamma }$$

where *t*_*s*_ = sintering or “healing” time, *a*_*0*_ = deposited trace radius of 0.25 mm, *η* = zero shear viscosity [Pa·s] based on the rheological measurements, and $\Gamma$ = surface tension of ABS at 30 mN/m^25^. Based on the sintering time calculated from Eq. (1), the fracture toughness *J*, is predicted with annealing time, *t*:

2 $$\frac{J-{J}_{0}}{{J}_{\infty }}=\left(1-{e}^{-t/{t}_{s}}\right)$$

where *J*_*0*_  is the  fracture toughness as printed, *J*_*∞*_  is the  maximum fracture toughness after complete annealing, and *t*  is the  annealing time. We propose two modifications to apply this model to the present data set. First, although the model is rigorously derived for fracture toughness, we hypothesize that Izod impact strength *K* will also be well modeled by this relationship. This hypothesis derives from the fact that the sintering model predicts contact area growth, which is presumed to be proportional to the Izod impact strength. Secondly, we wish to explicitly incorporate the effects of fill density. The limiting impact strength values *K*_*0*_ and *K*_*∞*_ are expected to scale with the contact area between layers which, for a simple 0–90 prismatic woodpile fill, scales with the square of the fill density (Supplementary Fig. S10a). Therefore, the impact strength is predicted to follow:

3 $$\frac{K-{v}_{f}^{2}{K}_{0}}{{{v}_{f}^{2}K}_{\infty }}=\left(1-{e}^{-t/{t}_{s}}\right)$$

where *K*_*0*_ and *K*_*∞*_ are Izod impact strengths for a 100% dense sample as printed, and fully annealed, respectively. To apply this model, we estimate that *K*_*0*_ = 2638 J/m^2^ and *K*_*∞*_ = 6847 J/m^2^, found by extrapolating from known as-printed and fully annealed values for samples at fill densities of 82% and 94% (Supplementary Fig. S10b).

**Figure 5:**
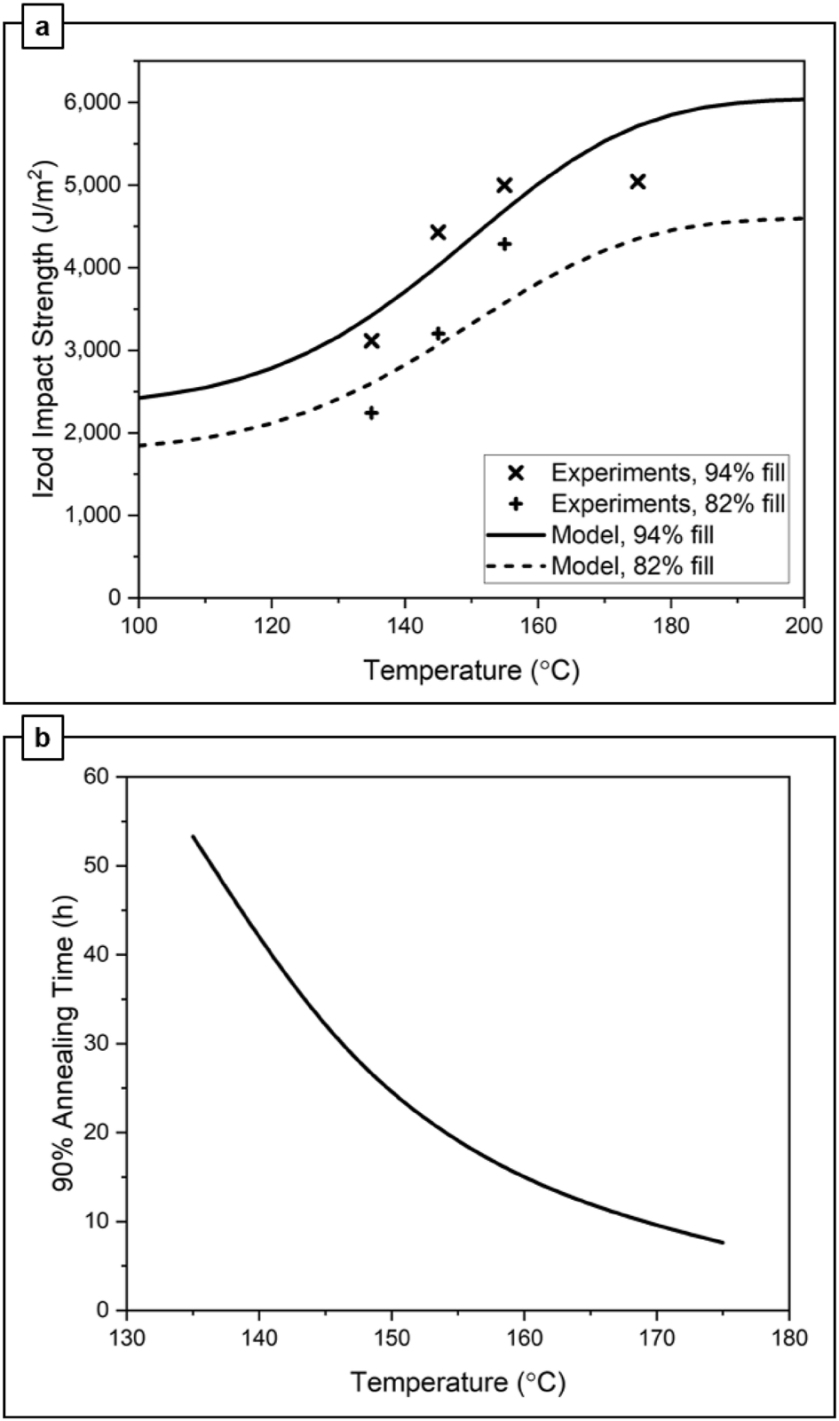
(**a**) Izod impact strength as a function of annealing temperature, after 8 h of annealing, and fill density (${v}_{f}$). The experiment data refers to the measured Izod impact strength from Fig. 2a. (**b**) Predicted annealing time necessary to achieve 90% of the fully annealed Izod impact strength, as a function of temperature.

Figure 5a uses Eq. (2) to show the predicted Izod impact strength with respect to temperature at 8 h annealing time; experimental data from Fig. 2a is plotted as a comparison. The annealing model reasonably captures the rise in impact strength with temperature, and the effect of fill density. The general agreement of model and experiment suggests that the degree of ABS wetting at print interlayers largely determines the Izod impact strength. Additional experimental data points would be useful to evaluate the model further. Figure 5b uses the annealing model of Eq. (3) to predict the time necessary to achieve 90% of fully annealed strength, as a function of temperature. This plot suggests that increasing the annealing temperature from 135 to 145 °C reduces annealing time from 53 to 30 h; increasing the annealing temperature to 155 °C reduces the required annealing time to only 18 h; and annealing at 175 °C would require only 8 h. Considering the excellent mechanical performance demonstrated in the present study after annealing for only 8 h at 155 °C, these modeled annealing time estimations may be conservative. However, the trends show the significant acceleration in annealing that can be achieved by increasing the annealing temperature.

Test results for the annealed samples in the camera mount adaptor engineering application (Fig. 4c) display two yield points. These yield points were correlated with cracks and whitening forming in two distinct areas of the part, where the first yield point is an interlayer crack, and the second is system failure at the bottom of the locking pin hole. These yield points are labelled 1 and 2 in Fig. 4c, d. This two-stage failure process was observed consistently in all annealed samples. In contrast, the as-printed samples consistently failed at a single crack in the interlayers surrounding the locking pin hole. These results show the importance of volumetric toughness, enabled by annealing, which allows yielding and load transfer during complex loading and failure. Images of the described failure behavior can be observed in Fig. 4d.

The selected 17–94 filament material pairing not only achieves superior annealing to the previous work Koker et al*.*^14^ by further closing the gap between free-standing annealed PC + ABS core–shell material and annealed in-mold ABS shell material, but also does so in just 11% of the time, at just 8 h as compared to the previous 72 h. This enables additively manufactured thermoplastic parts with enhanced toughness better suited for functional engineering applications which can be annealed overnight, such as the presented engineering application case study for a 3D printed tripod camera mount adapter. This work also highlights the effect of fill fraction, establishing its importance for both dimensional stability and maximizing Izod impact strength.

The current results demonstrate the thermal and mechanical stability of dual material filament. However, at present, the material combinations are limited to amorphous and compatible polymers. Future studies should explore the possibility of expanding the range of materials to include polymers with higher glass transition temperatures or semi-crystalline polymers like PEI or PEEK^7^ as core substitutes. Such expansion could enhance the resolution of existing dimensional stability and reduce the annealing time required, especially at higher temperatures. The upper bounds to making core–shell filament with a larger difference in T_g_ could be studied. Other mechanical properties could be investigated such as fatigue and tensile testing. Post or in-situ print processes such as heated chamber printing could lead to a reduction in annealing time.

## Methods

### Materials

The bicomponent filament core material was produced with two different grades of ABS, one PC, and one PC co-polymer. Stratasys (Eden Prarie, MN) ABS-M30 filament and PC-10 filaments were used to reproduce the core–shell filament from Koker et al^14^. Stratasys ABS and PC filament were pelletized to similar shape and size of the other ABS and PC grades prior to filament production. Covestro (Pittsburgh, PA) co-polymer polycarbonate grade APEC 1795 was chosen for its higher T_g_ of 173 °C compared to PC-10 (T_g_ = 144 °C). Sabic (Houston, TX) CYCOLAC MG94 extrusion grade ABS was chosen for its comparable viscoelastic, thermal, and bulk Izod impact strength (240 J m^−1^)^15^ to those of M30; results from our own rheological and thermal characterization of both ABS grades are presented in Supplementary Fig. S1. Any mechanical testing of mono PC-10 and ABS-M30 samples were printed using the original commercially available filament. APEC 1795, by itself, was not able to be printed due to its high melt temperature of 330 °C which exceeds our printer’s maximum nozzle temperature of 290 °C. Bicomponent filaments were evaluated in three combinations: PC-10 + ABS-M30, termed 10–30; PC-10 + ABS MG94, termed 10–94; and APEC 1795 + ABS MG94, termed 17–94 (Table 3). The results from these three combinations may provide insights for other material combinations with similar compatibility, morphology, and thermal properties, i.e., chemically compatible for blending, amorphous (as opposed to semi-crystalline), and high T_g_ core − low T_g_ shell.

**Table 3:** Filament combination naming scheme.

Material	Referenced As
Stratasys PC-10	PC-10
Stratasys ABS-M30	M30
Sabic CYCOLAC ABS MG94	MG94
Covestro 1795 PC	1795
PC-10 + M30	10–30
PC-10 + MG94	10–94
1795 + MG94	17–94

### Material characterization

Differential scanning calorimetry (DSC) was performed on a Mettler Toledo DSC 3+ (Greifensee, CH) to observe the glass transition temperatures of the ABS and polycarbonate grades, and the effect of reprocessing. An aluminum hermetic 40 µl pan and sample size of < 6 mg was used. The sample was heated at a rate of 10 °C /min from 25 to 250 °C for the first heating cycle, down to –10 °C then back to 250 °C. Nitrogen purge was introduced at a rate of 70 ml/min to avoid sample oxidation.

### Filament production

Thermoplastic filaments for this study were produced using a filament pilot line from Fibre Extrusion Technology (FET; Leeds, United Kingdom). Core–shell filament was fabricated in-house using the custom die presented in Koker et al*.*^14^. Mono-material filament was also fabricated in-house using a custom die suited to extrude diameter, *d*  = 1.75 mm filament.

The filament manufacturing set up was identical to that of Koker et al*.*^14^, with minor adjustments made run-to-run for better control of filament diameter and consistency between trials. Process conditions were selected referencing their respective material data sheet values^14–16^. The die pump zone was set to an intermediate value of the melt temperatures of the two materials. Materials were dried using a Dri-Air desiccant dryer (East Windsor, CT, USA) before processing. Process conditions for each filament combination can be found in Supplementary Table S1, in addition to further details about the custom dies in Supplementary Fig. S3.

The extruder melt pumps were calibrated for each material to achieve a mass ratio of 20% PC and 80% ABS. Extruded filament was drawn downwards into a Bay Plastics Machinery (Bay City, MI, USA) water bath, with a controlled water bath temperature of 50 °C. During the extrusion, the extrudate was dried using compressed air at exit from water bath and passed through a Metralight (Burlingame, CA, USA) micro XY laser micrometer, which measured two axes of the filament diameter at a sampling rate of 15 Hz. An example of this data for core–shell filament 17–94 is available in Supplementary Fig. S4. A Filabot (Barre, VT, USA) spooler at the end of the line provided adjustable tension used to achieve the desired filament diameter with a standard deviation of 0.014 mm or less.

### Specimen fabrication

All samples were printed on the Prusa MK3 (Prague, Czech Republic). Print settings were modified using Prusa slicer versions 2.0–2.4, outlined in Table 2. Prints took place within an enclosure fitted with a BOFA (Poole, United Kingdom) 3D PrintPRO 3 Fume Extraction System, an Eva-Dry (Tampa, FL, USA) renewable mini-dehumidifier, and a ThermoPro (Toronto, Ontario, Canada) moisture and temperature reader. Filament and printed samples were stored within a sealed desiccant box and maintained under 20% relative humidity. Print bed layout with sliced sample orientation can be found in Supplementary Fig. S5.

To achieve a high print density and avoid excessive voids and interlayer gaps, 12.7mm^3^ cubes were printed for each filament roll. The dimensions and mass of these cubes were measured, and the print density was calculated. The measured print mass was compared to the theoretical 100% bulk print mass to obtain the actual fill fraction. Then, the extrusion multiplier parameter in the print slicer software was adjusted up or down to control print fill fraction. This method is outlined in Eqs. (4) and (5), where ${v}_{f}$ is the actual fill fraction, ${m}_{theoretical}$ is the theoretical 100% bulk print mass, ${m}_{actual}$ is the measured mass of the print, and ${\rho }_{filament}$ is the density of the filament feedstock.

4 $${v}_{f}= 1- \frac{\left({m}_{theoretical}-{m}_{actual}\right)}{{m}_{theoretical}}$$

5 $${m}_{theoretical} ={\rho }_{filament}\cdot \left(l\cdot w\cdot h\right)$$

Two batches of test specimens were produced: one with ${v}_{f}$ = 82%, and one with ${v}_{f}$ = 94%.

Izod bars were printed following modified ASTM D256-10(2018)^17^, with sample geometries of 12.7 mm × 12.7 mm × 60 mm. Samples were printed vertically in sets of 5 then tested for Izod impact strength, with fracture directed through interlaminar material planes between build layers. Notching was performed one sample at a time with a Testing Machines Inc. notching cutter (Amityville, NY). Specimens were notched post-annealing to ensure dimensional accuracy of the notch. Annealed samples were notched on the top face as oriented during annealing.

“Benchy” refers to the nickname of a print file used as a benchmark assessment for the quality of a 3D printer^18^. These test articles were printed to observe the printability and thermal stability of the assessed filaments.

### Annealing procedures

All samples were annealed using Thermo Fisher Scientific (Waltham, MA) Heratherm General Protocol Oven. Izod bars were placed flat on an aluminum plate, perpendicular to the print orientation, on the long side. Mono-filament ABS samples were fixtured in an aluminum mold to maintain the geometry when annealed above the glass transition temperature. The oven was pre-heated to testing conditions before samples and their fixtures (see Supplementary Fig. S6) were inserted. All Benchy samples were annealed in air at 145 °C for 55 h.

### Mechanical testing

Izod tests were conducted to help identify relative impact strengths across each material pairing, and to further evaluate the best performing configuration. ASTM D256-10(2018)^17^ was followed, with a minimum of five samples per data set. Each sample’s mass and dimensions were recorded before and after annealing. Once notched using a single-toothed notch cutter (TMI Testing Machines, Model TM 22-05), Izod bars were individually tested with a Tinius Olsen (Horsham, PA) 503 digital impact tester.

Modified tensile testing was also performed using an Instron (Norwood, MA) 5966 Universal Testing System at a rate of 50 mm/min using a 10 kN load cell.

### Sample imaging

All cross sectional and fracture surface images were taken with a ZEISS (Oberkochen, Germany) optical microscope. For cross-sectional images, a diamond-blade precision saw (Buehler Isomet 1000) was used to achieve clean cuts and minimal pullout. Photos were taken of each precision-cut and Izod fracture surface using a combination of 1.0–3.5× objective lenses and up to 40× magnification.

### Supplementary Information

Supplementary Information.

## Data Availability

The authors declare that most data supporting the findings of this study are available within the paper and its supplementary information files. Additional data is available from the corresponding author upon reasonable request.
