# Supplementary material for: Accelerated annealing of fused filament fabricated (FFF) thermoplastics via an improved core–shell filament
Source: Sci Rep. 2023 Aug 19;13:13538. doi: 10.1038/s41598-023-40855-6 (PMC10439886; doi:10.1038/s41598-023-40855-6)
Supplement: Supplementary file 1 — Supplementary Information. [file 41598_2023_40855_MOESM1_ESM.docx]

**Supplementary Information**

**Accelerated annealing of fused filament fabricated (FFF) thermoplastics via an improved core-shell filament**

**Michael Pugatch^1^*, Molly Teece^1^*, Juhyeong Lee^1^, Nikhil Patil^1^, Ryan Dunn^2^, Kevin Hart^3^, Eric Wetzel^2^, and Jay H. Park^1**^**

^1^University of Massachusetts-Lowell, Department of Plastics Engineering, Lowell, 01854, USA

^2^United States Army Research Laboratory, Aberdeen Proving Ground, MD 21005, USA

^3^Milwakuee School of Engineering, Milwaukee, WI 53202, USA

*These authors contributed equally to this work

**Corresponding author: [Jay_Park@uml.edu](mailto:Jay_Park@uml.edu)

**Rheology**

*Experimental Procedure*

Parallel plate rheology was performed to evaluate the viscoelastic properties of the Cycolac MG94 polymer. Polymeric discs 20 mm in diameter and approximately 1.5 mm thick were compression molded from small pellets of as-received polymer in an oven at 150 °C under 1 atm of pressure.

Rheological measurements were performed on a Discovery HR-2 rheometer (TA Instruments; New Castle, DE). Parallel plates were made of aluminum and had a diameter of 25 mm. The gap between plates was kept constant at 1 mm during testing. Isothermal frequency sweeps were executed between frequencies of 0.01 and 10 Hz at temperatures of 170, 180, 190, 200, and 210 °C at a shear strain of 1.0% with 10 minutes of equilibration time between each temperature setting. To begin testing, plates were heated to 210 °C and held at temperature for 10 minutes. Polymer discs made during compression molding were then placed between the heated parallel plates before plates were closed to a 1 mm gap. Excess polymer was trimmed from the plates and the testing was initiated. Testing of the same sample at subsequent temperatures immediately followed until all isothermal tests were completed.

*Results*

The master curve of the storage modulus as a function of frequency is plotted in Figure S1a alongside calculated shift factors in **Figure S1b**. Shift factors were fitted using the Williams-Landel-Ferry (WLF) model^[23]^:

$$\log_{10} \left( \alpha\right)=-\frac{C_{1}\left( T-T_{ref} \right)}{C_{2}+\left( T-T_{ref} \right)}$$

At a reference temperature of 190 °C. Constants $C_{1}$ and $C_{2}$ were found to be 4.48 and 172.3 °C respectively. These constants for the WLF model of shift factor for Cycolac MG94 very closely mimic those of the ABS M30 material^[13]^ of 5.09 and 166.9 °C, indicating very similar rheological behavior between these two materials.


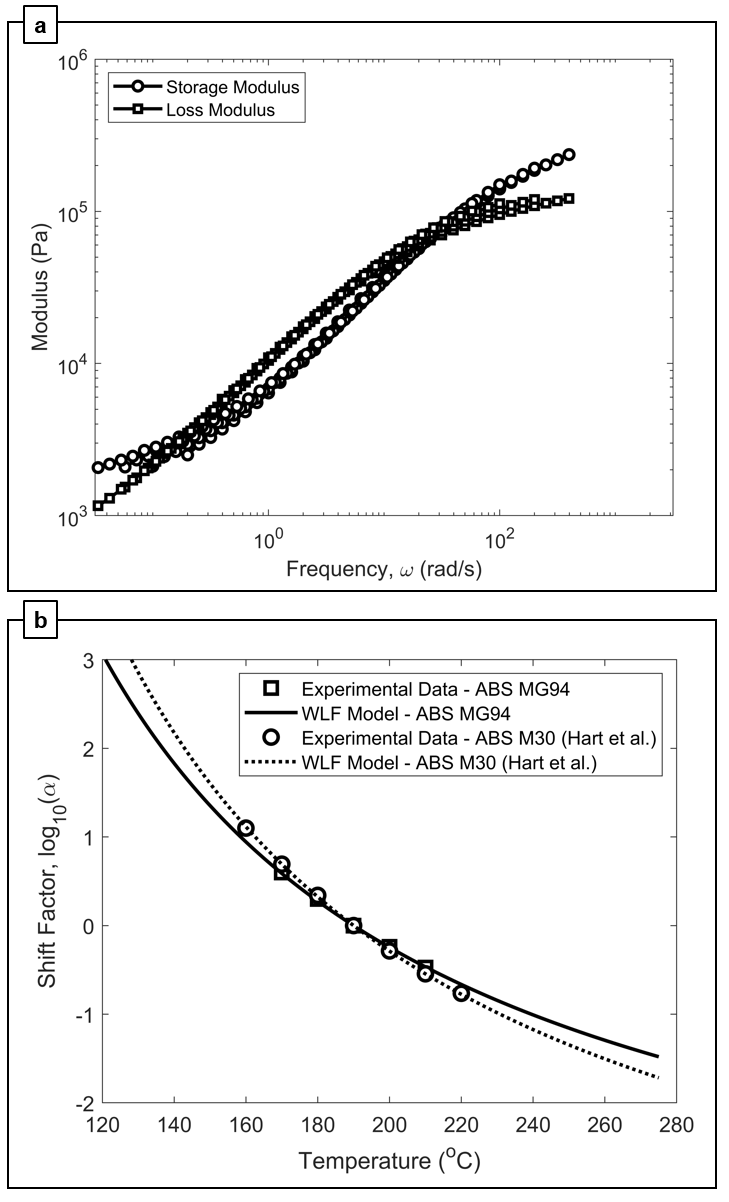


**Figure S1**. Time-temperature superposition rheological models of ABS polymers M30 and Cycolac MG94. (a) Master curve of storage and loss moduli at a reference temperature of 190 °C for Cycolac MG94 ABS. (b) Shift factors from time-temperature superposition analysis of Cycolac MG94 (current work) and ABS M30^[13]^. Experimental data in (b) is fit using the Willliams-Landel-Ferry (WLF) model^[23]^.

**Differential Scanning Calorimetry**

*Experimental Procedure*

Differential scanning calorimetry (DSC) was employed to compare critical thermal transitions in both the Cycolac MG94 and ABS M30 polymers. Testing was performed in accordance with ASTM D3418. Dynamic heat-cool-heat cycles were performed separately on as-received pellets of Cycolac MG94 and on small segments of ABS M30 filaments cut from an as-received spool. For each sample type, approximately 10 mg of material was placed into an aluminum DSC pan, then hermetically sealed. Samples were initially held at 40 °C for 5 minutes, then underwent a heat-cool-heat cycle between temperatures of 40 and 200 °C at a rate of 5 °C/min while heat flow was recorded. Measurements were performed on a Q1000 DSC (TA Instruments; New Castle, DE).

*Results*

Results of dynamic heat-cool-heat DSC testing are provided in Figure S2. Heat flow traces for both polymers are nearly identical, showing endothermic heat absorption at a temperature of 105 °C which is the listed glass transition temperature of these polymers. Also notable is the lack of any crystallization or melt peaks for both materials. In terms of critical polymeric transitions and amorphous character, the MG94 and M30 materials are nearly indistinguishable using calorimetry.


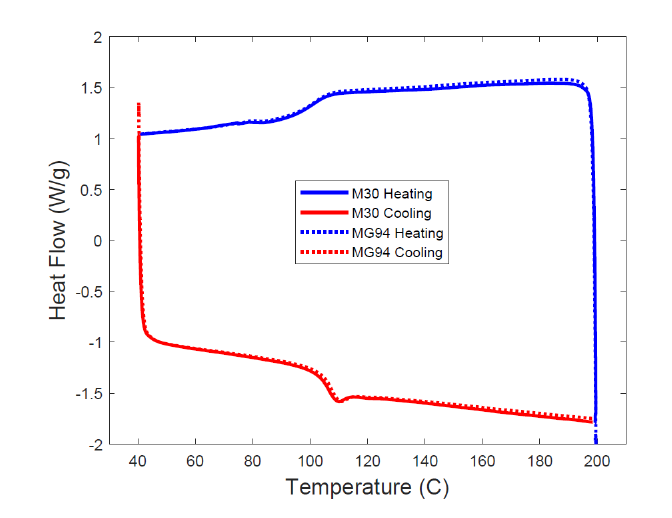


**Figure S2**. Heat flow as a function of temperature (exo up) for ABS M30 and Cycolac MG94 ABS materials observed during dynamic scanning calorimetry.

**Table S1**. Process conditions for different core-shell filament combinations with FET

| **Material Pairing** | **Material Grade** | **Melt Pump Speed [rpm]** | **Zone 1 [°C]** | **Zone 2 [°C]** | **Zone 3 [°C]** | **Zone 4 [°C]** | **MPZ [°C]** | **DPZ [°C]** |
| --- | --- | --- | --- | --- | --- | --- | --- | --- |
| 10-30 | Stratasys PC-10 | 6 | 225 | 240 | 250 | 260 | 260 | 250 |
|  | Stratasys M30 | 12 | 205 | 220 | 230 | 240 | 240 |  |
| 10-94 | Stratasys PC-10 | 6 | 225 | 240 | 250 | 260 | 260 | 250 |
|  | Cycolac MG94 | 12 | 205 | 220 | 230 | 240 | 240 |  |
| 17-94 | APEC 1795 | 6 | 295 | 310 | 320 | 330 | 330 | 285 |
|  | Cycolac MG94 | 16 | 205 | 220 | 230 | 240 | 240 |  |


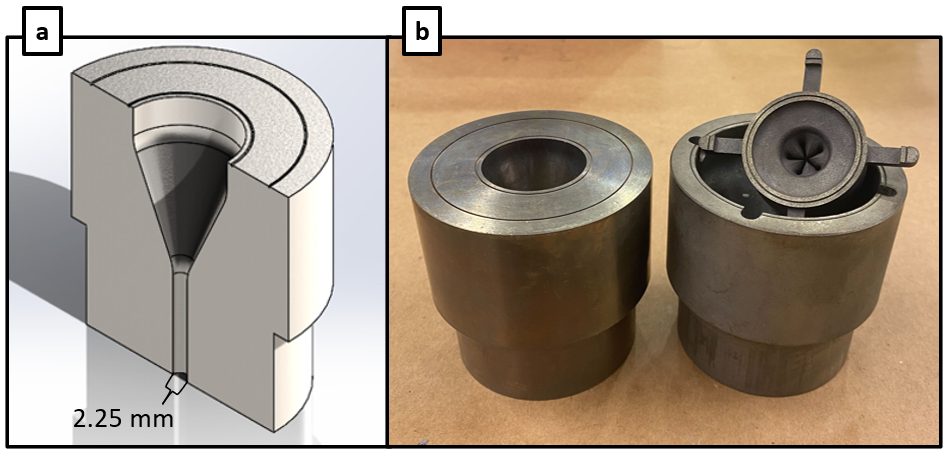


**Figure S3**. (a) Solidworks cross section of mono component die (b) Machined mono die (left) with custom asterisk core-shell die for filament production


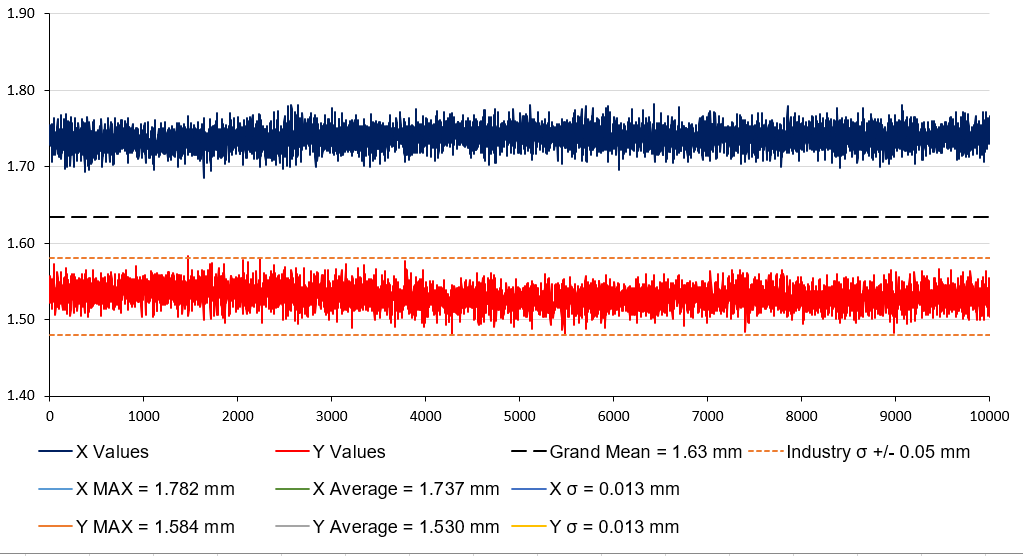


**Figure S4**. XY micro laser micrometer data for 17-94 filament spool production


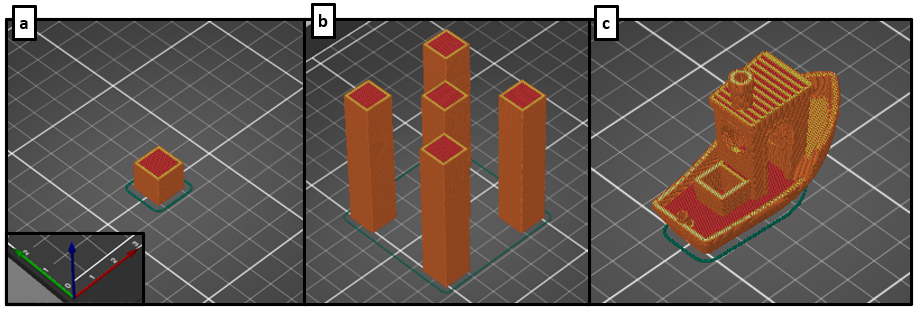


**Figure S5**. Prusa slicer software MK3 print bed’s layout of each sample. Located in the center, sliced models of (a) calibration cube (b) Izod bars (c) Benchy


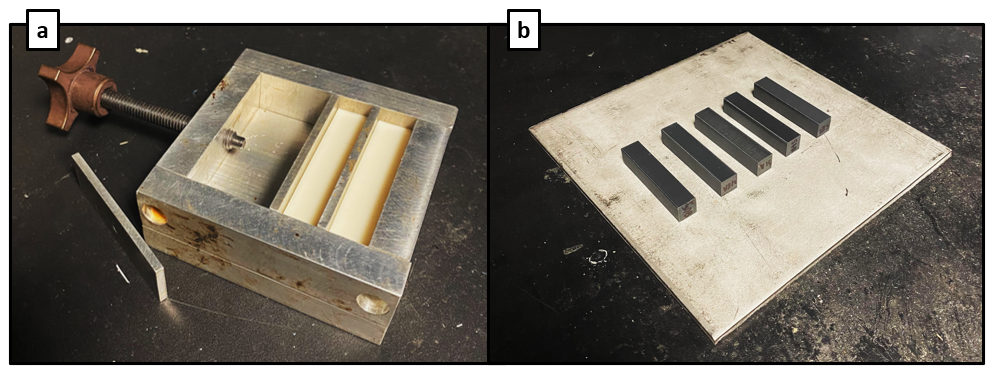


**Figure S6.** (a) ABS Izod specimen annealing fixture (b) Izod specimen annealing plate for PC+ABS core-shell specimen


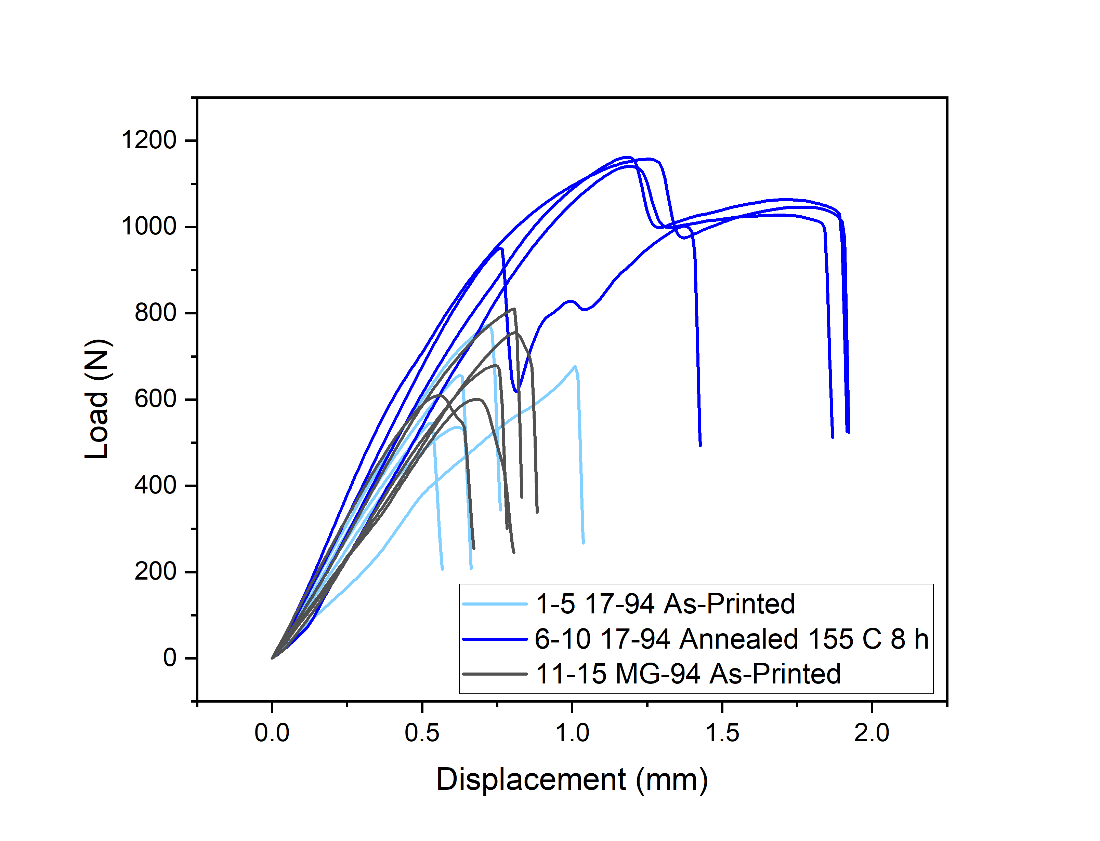


**Figure S7**. Complete tensile data of engineering application


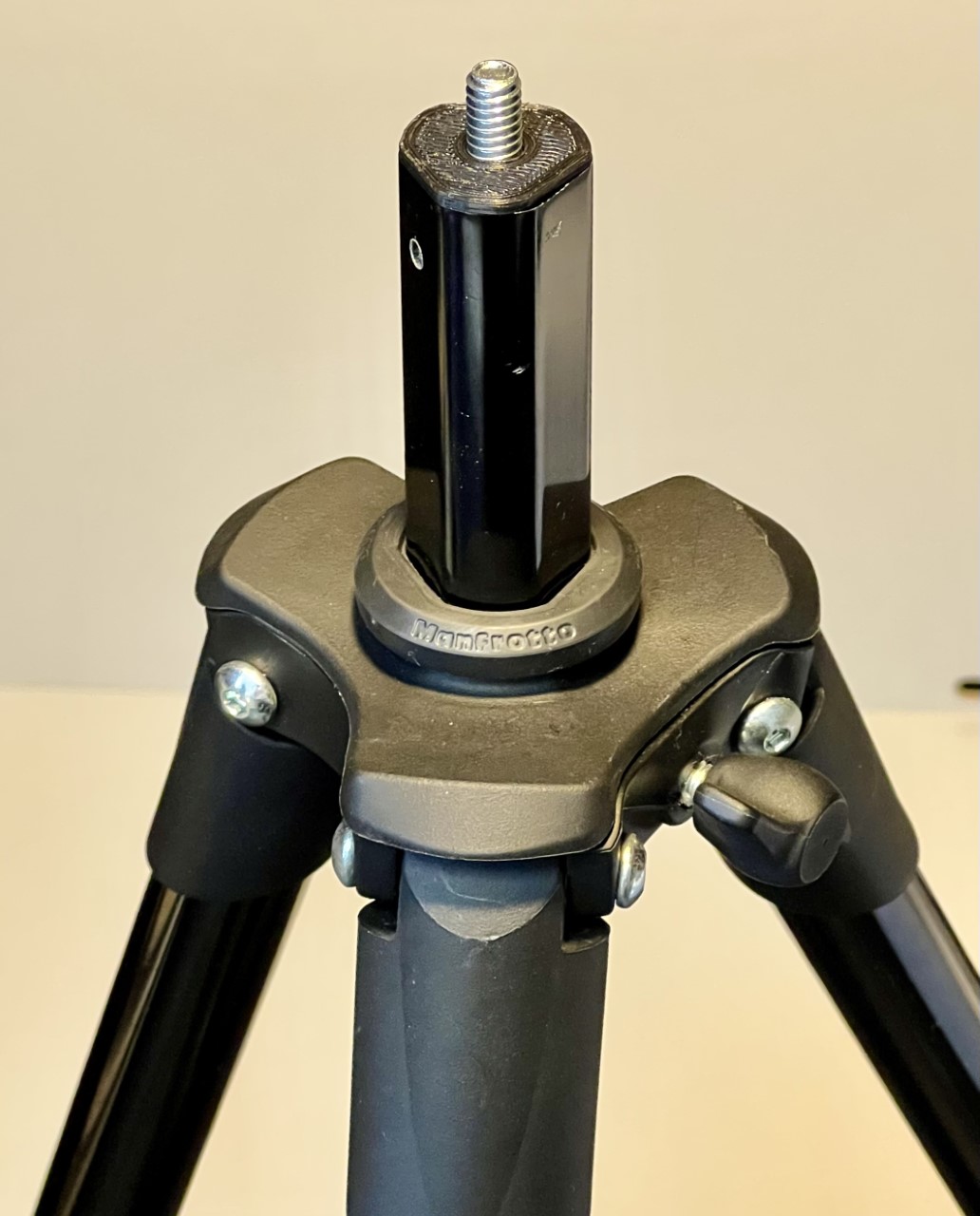


**Figure S8.** Tripod assembled with adaptor. The adaptor enables standard accessories compatible with the bolt to be attached.


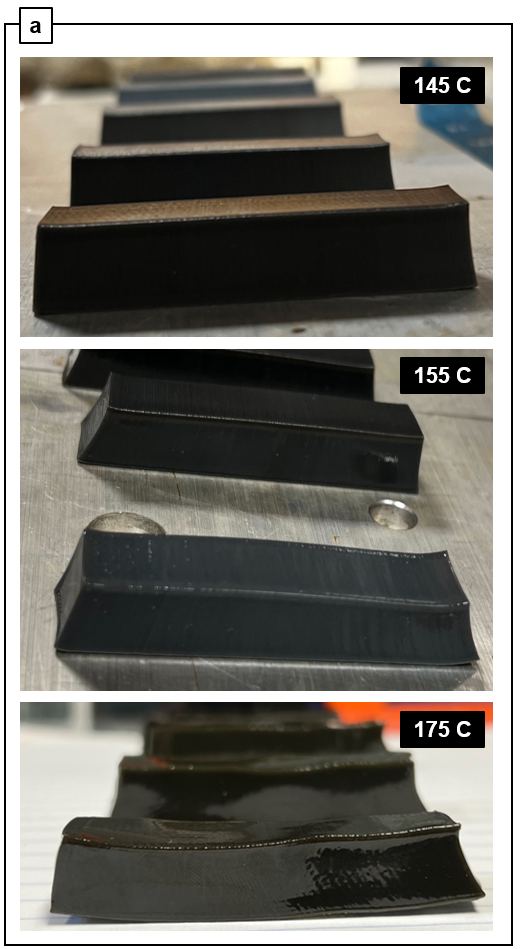


**Figure S9**. Annealed 17-94 Izod bars at 145 °C, 155 °C, and 175 °C, for 8 h at $v_{f}$ = 82%, which display poorer dimensional stability as temperature increases. As described in the main texts, $v_{f}$ = 94% samples exhibited superior dimensional stability after annealing (not pictured here).

**Annealing model**

Our modified annealing model assumes that the as-printed and fully annealed Izod impact strength values will scale with the fill density, $v_{f}$, squared. This scaling hypothesis is based on the geometry shown in Fig. S10a, representative for a 0-90 fill pattern FFF part. The fill density, or solid volume fraction, for each layer is given by the trace width *b* divided by the center-to-center spacing of the traces *w*. The contact area between layers, normalized by the total area between layers, is (*b*/*w*)^2^. Therefore, the contact area between layers should be proportional to *v_f_*^2^. Since Izod impact strength should be proportional to the total bonded area through which a fracture is driven, then it is reasonable to hypothesize that Izod impact strength will also be proportional to *v_f_*^2^.

This scaling can be implemented in the impact strength model, if the as-printed and fully annealed Izod impact strength values, *K_0_* and *K_¥_* , are known for a fully dense (*v_f_* = 100%) part. The present experiments did not include fully dense parts, so instead we use the available data to estimate these values. First considering *K_0_* , Fig. S10b plots *K_0_* as a function of *v_f_*^2^. A data point of zero impact strength at zero fill density is also included as a likely lower limit. The three data points are well fit (R=0.9999) by a line with a zero *y*-intercept, suggesting that the *v_f_*^2^ scaling is appropriate. Extrapolating forward gives a *K_0_* value for a fully dense part of 2638 J/m^2^. For *K_∞_*, our only available data point is for a 94% dense part composed of MG94 (no PC core), which gave a fully annealed (8 h at 135 °C) Izod impact strength of 6050 J/m^2^. Using zero impact strength at zero fill density as the other data point to define a line versus *v_f_*^2^, the forward extrapolated value for a fully annealed, fully dense part is *K_∞_*= 6847 J/m^2^ . These values for *K_0_* and *K_∞_*, for parts with *v_f_* = 1, are used in the annealing model for predicting Izod impact strength under intermediate states of fill density and annealing.


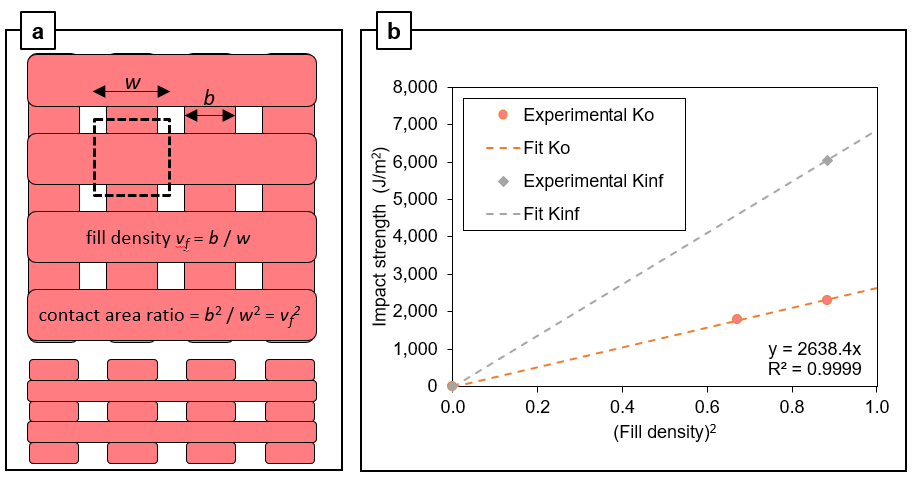


**Figure S10.** (a) Schematic of overlap area in underfilled, 0-90 fill pattern FFF part. (b) Experimental data points for Izod impact strength versus fill density (*v_f_*) squared; and fit to each set of data.
